# Endoscopic full-thickness resection using an over-the-scope device for treatment of recurrent / residual colorectal neoplasia: a single-center case series

**DOI:** 10.1186/s12876-019-1043-8

**Published:** 2019-07-10

**Authors:** Annabell von Helden, Ralf Hildenbrand, Bernd Sido, Franz Ludwig Dumoulin

**Affiliations:** 1 Department of Medicine and Gastroenterology, Gemeinschaftskrankenhaus Bonn, Academic Teaching Hospital, University of Bonn, Bonner Talweg 4-6, 53113 Bonn, Germany; 2 Institute for Pathology Bonn-Duisdorf, Heilsbachstr. 15, 53123 Bonn, Germany; 3 Department of General and Abdominal Surgery, Gemeinschaftskrankenhaus Bonn, Academic Teaching Hospital, University of Bonn, Bonner Talweg 4-6, 53113 Bonn, Germany

**Keywords:** Colorectal neoplasia, Endoscopic full thickness resection, Adenoma recurrence, Fibrosis, Perforation, Bleeding

## Abstract

**Background:**

Endoscopic mucosal resection (EMR) in piecemeal technique is the treatment standard for larger flat or sessile colorectal lesions. The method is burdened by a high recurrence rate mostly presenting as difficult to resect lesions. In these situations, endoscopic full thickness resection (EFTR) with an over-the-scope device offers the option of complete resection despite scar formation.

**Methods:**

We conducted a retrospective case review of 30 consecutive EFTR interventions on small (< 20 mm), difficult to resect recurrent / residual colorectal neoplastic lesions treated by EFTR.

**Results:**

EFTR was technically feasible in 28/30 (93,3%) of the cases with an R0 resection in 24/30 (80%) and a median procedure time (marking to full thickness resection) of 34,5 min (11–120). After the first 15 procedures, the per-protocol rate increased from 13/15 to 15/15 and the R0 resection rate increased from 9/15 (69,2%) to 15/15 (100,0%; *p* < 0.01). One patient suffered from a delayed perforation the day after the procedure and needed emergency surgery (3,6%). Minor bleeding occurred in 3/28 patients (10,7%) and post-interventional fever in one patient (3,6%). The 30-day mortality rate was 0%.

**Conclusions:**

EFTR with an over-the-scope device is a useful method for endoscopic resection of difficult to treat recurrent or residual colorectal neoplasia after previous endoscopic therapy. High R0 resection rates were observed after a relatively short learning curve. The complication rate in this series seems acceptable given the complexity of the resected lesions.

## Background

Polypectomy and endoscopic mucosal resection (EMR) - either en bloc or in piecemeal fashion – are established treatment standards for colorectal neoplasia such as adenoma or low risk early cancer [1–3]. Problems arise in cases of incomplete resections after fragmented (so called ‘piecemeal’) mucosectomy with reported recurrence rates of 20–30% [4, 5]. In fact, incomplete polypectomy probably has a relevant impact on interval cancers [6]. While recurrences after previous endoscopic therapy are usually small, their treatment is difficult due to scar formation. Even endoscopic submucosal dissection (ESD) - favored by Japanese guidelines [7] - does not reliably result in R0 resections in cases of severe fibrosis [8, 9].

Recently, an endoscopic full-thickness resection (EFTR) device (FTRD®, Ovesco, Germany) has been developed. The device combines a large over-the-scope-clip (OTSC) to create a full thickness plication with a snare, which is subsequently used to cut the full thickness specimen [10, 11]. The method has been applied for the treatment of colorectal non-lifting lesions, lesions in anatomically difficult localization and also for smaller subepithelial tumors in several case series and one large prospective multicenter study [12–19]. In this study, we have focused on the clinically important aspect of endoscopic treatment for difficult to resect colorectal neoplasia after previous endoscopic therapy.

## Methods

### Patients and lesions

Between 01/2016 and 11/2018 we performed 30 endoscopic full thickness resections using the FTRD® device (Ovesco, Tübingen, Germany) in 29 consecutive patients (19 male / 10 female; median age 72,7 years / range 21,5-81,6; Table 1). All lesions were < 20 mm in size and were judged difficult to resect because of non-lifting due to scar formation after previous endoscopic resection. Localization of the lesions was in the right colon (*n* = 18), left colon (*n* = 9) and rectum (*n* = 3). The procedure time was measured from the initial marking of the lesion until the final endoscopic control of the clip closure. All procedures were performed by a single endoscopist (F.L.D.) who had received team training with the FTRD® device on isolated pig colon provided by the manufacturer. Informed consent for the procedure was obtained from all patients. Data on endoscopic procedures were prospectively recorded (Clinic WinData, E&L, Erlangen, Germany). Retrospective analysis was done by review of the digital patient charts. The study conforms to the provisions of the Declaration of Helsinki. It was approved by institutional board review of the Gemeinschaftskrankenhaus Bonn and - in accordance with federal regulations (§ 15 of the professional code / ‘Berufsordnung für die nordrheinischen Ärztinnen und Ärzte’) - specific consent to participate in this retrospective case series was waived.

**Table 1:** Patients and lesions

Patients (*n* = 29) and lesions (*n* = 30)	
Age, years (median / range)	72,7 (21,5-81,6)
Gender (female / male)	10 / 19
Localization (n)	
• right colon (ascending / transverse)	18
• left colon (descending / sigmoid)	9
• rectum	3
Pre treatment histology (n)	
• serrated adenoma	2
• tubular or tubular-villous adenoma; low grade IEN	16
• tubular or tubular-villous adenoma; high grade IEN	8
• carcinoma	4
Procedure time, marking to final control (median / range)	34,5 (11-120 min)
Size of resected specimen, max. diameter (median / range)	25 (14-33 mm)

### EFTR procedure with the FTRD device

**Fig. 1:**
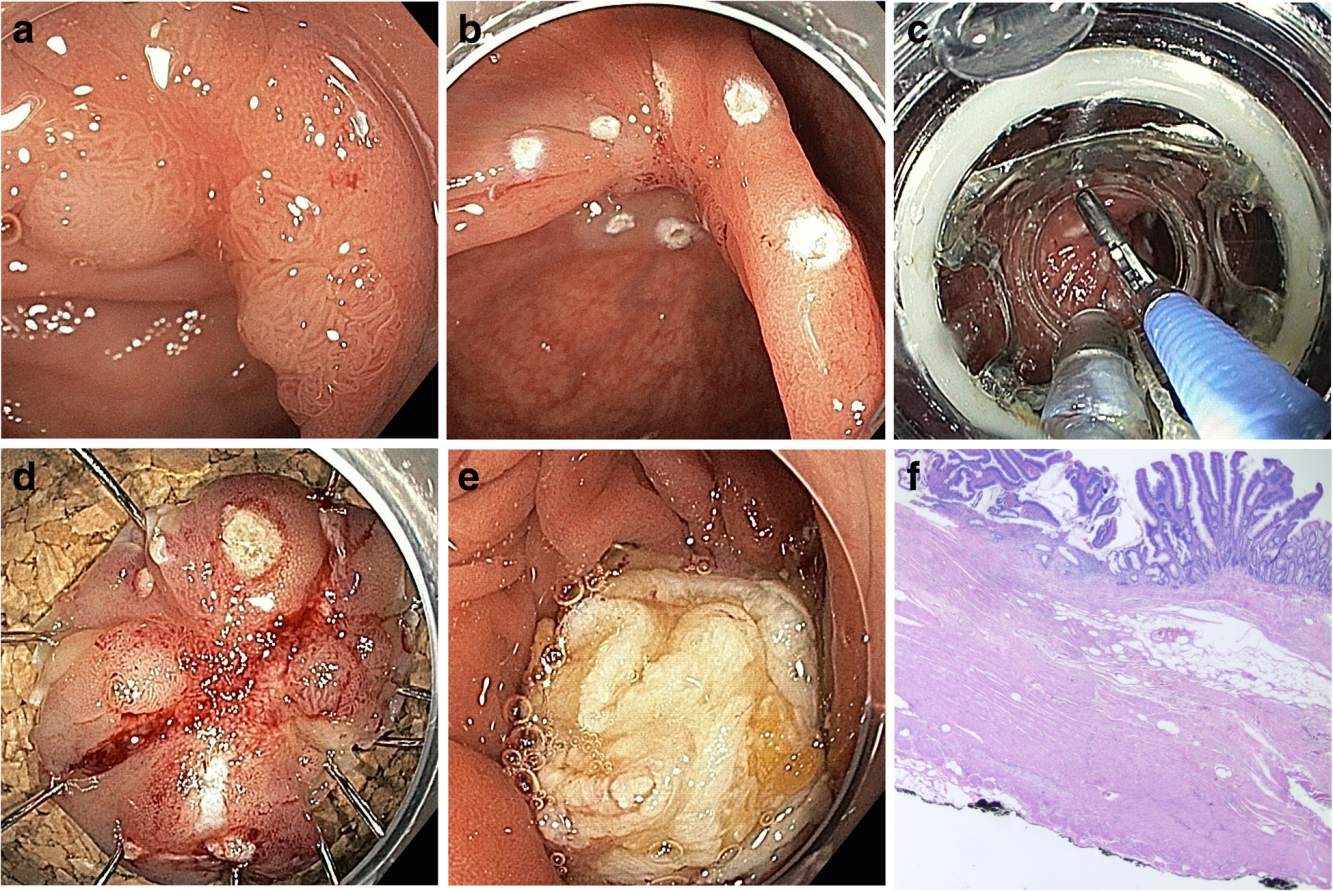
Endoscopic full thickness resection with the FTRD® device. **a** Residual adenoma after previous piecemeal EMR; note the central scar. **b** Marking of the lesion. **c** Retraction of the lesion into the FTRD cap. **d** Resected specimen pinned on corkboard. **e** Resection site with FTRD clip in situ. **f** Histopathology showing full thickness resection of adenoma

EFTR was carried out under conscious sedation with propofol, fentanyl and prophylactic single shot antibiosis (cefuroxime). After endoscopic evaluation and marking of the lesion (Fig. 1a, b), the FTRD® system was mounted and the endoscope re-advanced to the lesion. Full thickness resection with the integrated snare was accomplished after pulling the tissue into the cap and release of the clip (Fig. 1c, d). The settings of the Erbe Vio 3 electrosurgical unit (Erbe Elektromedizin, Tübingen, Germany) were forced coagulation 1.0 for initial marking of the target lesion and endocut Q 1.0 for cutting the lesion. After retrieval of the specimen and removal of the FTRD® system a repeat endoscopy was performed to control the positioning of the clip (Fig. 1e) and to rule out any possible injury that might have happened during the passage of the FTRD® device to the target lesion.

### Histopathology

The specimens were gently stretched and then fixed on corkboard. Histopathology was performed with specific attention to the resection margins to confirm a complete resection (Fig. 1f). Patients with early cancer were presented in the interdisciplinary tumor board.

### Post-procedural care

Patients had a liquid diet for the first 6 h after the procedure and were allowed to consume a soft diet the day after. We performed clinical visits and laboratory controls on the evening after the procedure as well as on the first post-interventional days and in any case of suspected adverse events. The majority of patients could be discharged on the second post-interventional day. We recommended follow up endoscopies in accordance with the current German S3 guideline [3].

### Definition of outcome and complications

We defined ‘technical feasibility’ as reaching the target lesion and ‘R0 resection’ if both vertical and lateral margins were free of adenoma or cancer. A ‘perforation’ was diagnosed if a complete transmural lesion was identified during the procedure or if clinical and radiological signs of perforation were demonstrated after the procedure. A ‘major bleeding’ was defined as a loss of ≥3 hemoglobin units with signs of gastrointestinal bleeding after the completion of the intervention.

### Statistics

Group comparisons were done with Fisher’s exact test for categorical (https://www.socscistatistics.com) and students t-test (Microsoft Excel for Mac 2011) for continuous variables.

## Results

### Efficacy

**Fig. 2:**
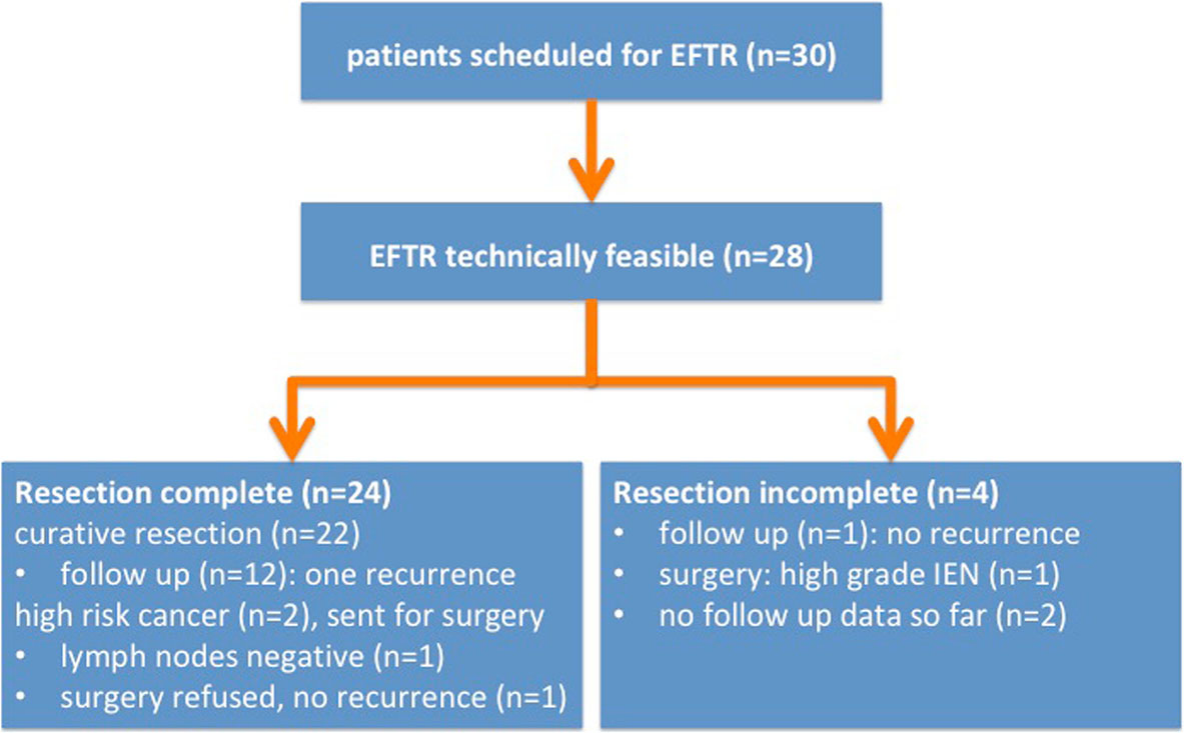
Outcome in 30 EFTR procedures. Flowchart with clinical outcome in 30 EFTR procedures

EFTR was technically feasible in 28/30 patients (93,3%) with a median procedure time (marking to full thickness resection) of 34,5 min (range 11–120). In two patients the FTRD device could not be advanced to the lesion due to sigmoid (inflammatory) strictures. Both full thickness and R0 resection rates were of 80% (24/30) on intent to treat analysis and 85,7% (24/28) on per protocol analysis (Fig. 2, Table 2).

**Table 2:** Outcome of 30 EFTR procedures

	Number	Intent to treat (*n* = 30)	Per protocol (*n* = 28)
Full thickness resection	24	80,0%	85,7%
R0 resection	24	80,0%	85,7%
Complications			
• perforation	1	3,3%	3,6%
• minor bleeding	3	10,0%	10,7%
• fever after EFTR	1	3,3%	3,6%
• emergency surgery	1	3,3%	3,6%
• 30-day mortality	0	0,0%	0,0%

In four EFTR interventions we could not achieve R0 resection. The endoscopic resection was judged complete in two of them, but histology showed positive resection margins. Follow up has been done for one patient and did not show residual neoplasia. In two other patients we had experienced difficulties in mobilizing the colonic wall into the resection cap due to fibrosis. The resection was diagnosed as incomplete at the final endoscopic control of the resection area. One patient had additional surgery due to high grade intraepithelial neoplasia; follow up data are not available for the other (Fig. 2).

The final histology showed advanced lesions in 12 patients including four carcinomas that had been diagnosed beforehand for possible curative treatment (Table 3). Two of these were evaluated as low risk lesions and - according to the German S3 guideline [3] – the patients were left without additional surgery. The two other lesions had been classified as potentially deep submucosal invasive according to the NICE classification [20] and the patients had been informed about the possibility of a non-curative resection. Histopathology showed pT2, G2, Pn0, L0, V0 - R0 in both cases and surgery was recommended. No lymph node metastasis was found after surgical resection in one patient; the other refused additional treatment and is without recurrence or metastasis after 14 months of follow-up.

**Table 3:** Final histology of 28 resected specimens

	Number	Percentage
Serrated adenoma	2	7,1%
Tubular or tubular villous adenoma, low-grade IEN	14	50%
Tubular or tubular villous adenoma, high-grade IEN	8	28,6%
Carcinoma, low-risk	2	7,1%
Carcinoma, high-risk	2	7,1%

Endoscopic follow-up data are available for 12 patients with one adenoma recurrence despite previous R0 EFTR resection after intial piecemeal EMR of a large rectal lesion (Fig. 2).

### Complications

We observed one major complication, a delayed perforation the day after EFTR necessitating emergency surgery. In addition, three patients reported minor, self-limiting hematochezia and one patient developed fever without abdominal pain after EFTR. The 30-day mortality rate was 0% (Table 2).

### Learning curve

When analyzing the efficacy and complication rates of EFTR procedures a significant increase in R0 resection rate was observed after the first 15 procedures (9/15 vs. 15/15; *p* < 0.01). Procedure times were not significantly decreased: median 36 min (range 21–120) versus 31 min (range 11–69). Of note, the two cases where the FTRD device could not be advanced to the lesion were among the first 15 procedures.

## Discussion

To date, there are limited data on endoscopic full thickness resection with the FTRD device including one large prospective study treating both epithelial and subepithelial lesions [15–19]. While these studies mainly concentrated on technical aspects, including EFTR for different indications, this study focused on EFTR procedures with the FTRD device in patients with difficult to treat, non-lifting residual / recurrent neoplasia after previous endoscopic resection. Indeed, this is a very common problem, giving the fact that fractionated mucosectomy is the standard treatment for larger flat or sessile colorectal neoplasia in western countries. We observed two treatment failures, were the device could not be advanced to the target lesion. For the remaining 28 procedures the R0 resection rate was 85,7%. A relatively quick learning curve was observed for the R0 resection rate, which increased from 9/15 to 15/15. Emergency surgery was needed in one patient when perforation developed the day after the procedure.

The efficacy data presented here are comparable to data from the largest prospective study with the FTRD device published [15] and two other smaller case series [16, 17, 19] (Table 4). They are also comparable to results of ESD in these particular situations [7]. However, compared to other advanced endoscopic resection techniques such as ESD [21], the method has a quick learning curve, which lead to an R0 resection of 100% after having performed the first 15 cases.

**Table 4:** Outcome of studies using the FTRD device

Study, year	(n)	Lesions (n)	Technical feasibility	Full thickness	R0	Emergency surgery
Richter-Schrag, 2016 [17]	20	recurrence (9) non-lifting (3) T1 Carcinoma (6) NET (2)	75%	80%	80%	5%
Schmidt, 2018 [15]	181	recurrence (72) non-lifting (32) T1 Carcinoma (15) SET (23) difficult location^a^ (39)	89,5%	76,9%	76,9%	2,2%
Valli, 2018 [18]	60	recurrence (29) T1 Carcinoma (6) SET (5) difficult location (5) other^b^ (15)	88%	88,8%	79%	2,0%
Vitali, 2018 [16]	13	recurrence (4) non-lifting (7) NET (1) difficult location (1)	100%	n.a.	83,3%	0,0%
Andrisani, 2019 [19]	110	recurrence (65) non-lifting (12) T1 Carcinoma (16) SET (10) difficult location (4) other (3)	94,4%	91,0%	92%	n.a.
This study	30	recurrence (30)	93,3%	80,0%/ 85,7%	80,0%/ 85,7%	3,3%/ 3,6%^c^

^a^Difficult location: e.g. at the appendix or a diverticulum

^b^Other: e.g. in addition to piecemeal resection; after resection of malignant polyp

^c^Data for R0 resection and emergency surgery given as intent to treat / per protocol, respectively.

The specific advantage of full thickness resection became obvious in the case of the two high-risk cancers. In fact, these lesions were already pT2 cancers with initial infiltration of the proper muscle layer. In this situation EMR or ESD might have resulted in perforation and potential translocation of cancerous tissue – which was avoided by full thickness R0 resection. On the other hand it must be emphasized, that even R0 resection of a recurrence does not rule out further recurrence at the same resection site. Thus, one patient developed a second recurrence despite initial R0 resection of a first recurrence. It is therefore mandatory to encourage timely endoscopic follow up – even after R0 resection of a recurrent adenoma – in particular if recurrence occurs after fragmented resection of a very large adenoma.

The rate of emergency surgery reported here is relatively high compared to other EFTR studies [14–16, 18] or to studies on EMR or ESD [1, 3, 7]. We think this is due to the fact that the study included only a relatively small number of cases. Moreover, no perforation occurred after the first 15 interventions. Finally, it must be pointed out, that EFTR was performed on lesions that otherwise would have been treated either by coagulation (i.e. no control over completeness of eradication) or sent for laparoscopic surgery.

## Conclusion

In conclusion, EFTR with the FTRD device is a useful tool for smaller sized difficult-to-resect colorectal neoplasia e.g. after previous piecemeal resection. After a short learning curve high R0 resection rates are observed. The rate of emergency surgery is substantial but seems acceptable given the complexity of the resected lesions.

## Data Availability

The datasets used and/or analysed during the current study are available from the corresponding author on reasonable request.

## Abbreviations

EFTR: Endoscopic full thickness resection
EMR: Endoscopic mucosal resection
ESD: Endoscopic submucosal dissection
FTRD: Full thickness resection device
G: Grading
IEN: Intraepithelial neoplasia
L: Lymphatic infiltration
n.a.: not available
NET: Neuroendocrine tumor
Pn: Perineural infiltration
pT: primary tumor
R: Resection status
SET: Subepithelial tumor
sm: submucosal
V: Vessel infiltration
